# The supramolecular chemistry of monodisperse 1,3,5-triazine oligomers

**DOI:** 10.1039/d5ob00321k

**Published:** 2025-03-17

**Authors:** Luis Escobar, Christopher A. Hunter

**Affiliations:** a Yusuf Hamied Department of Chemistry, University of Cambridge Lensfield Road Cambridge CB2 1EW UK herchelsmith.orgchem@ch.cam.ac.uk

## Abstract

Organic compounds based on 1,3,5-triazine scaffolds are utilised in practical applications in agriculture, as well as in the pharmaceutical and plastic industries. In fundamental research, 1,3,5-triazines are used as building blocks for the construction of oligomers that are relevant to the areas of supramolecular chemistry, chemical biology and polymer science. Here, we review the molecular recognition and self-assembly properties of monodisperse linear and branched oligomers, macrocycles, and dendrimers of 1,3,5-triazine. We focus mainly on experimental studies conducted in solution, describing the key interactions and structural features of these systems.

## Abstract 2

This review describes the molecular recognition and self-assembly properties of 1,3,5-triazine-based oligomers of different topology: linear, branched, macrocyclic, and dendrimeric.

## Graphical abstract

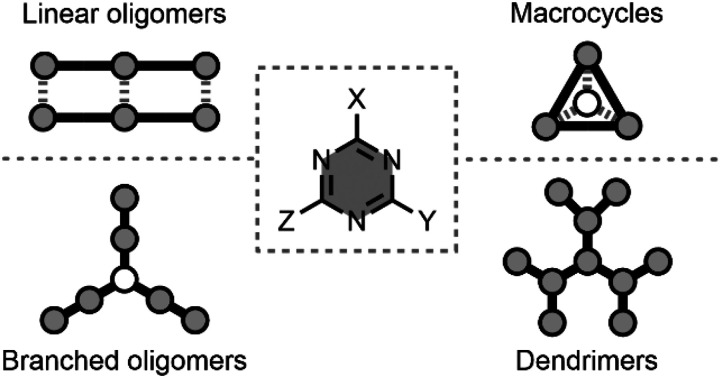

## 1. Introduction

**Fig. 1:**
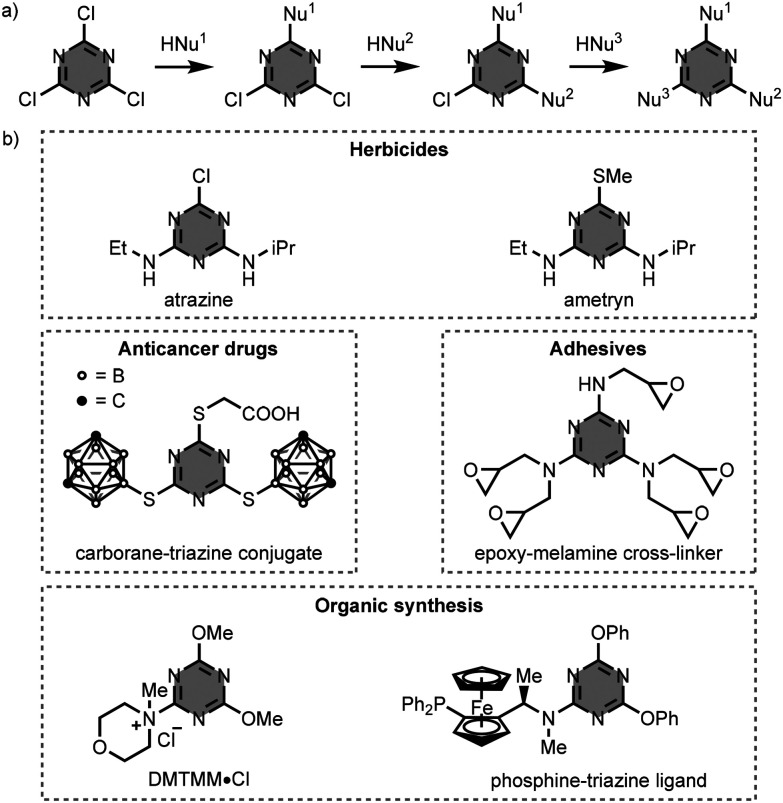
(a) General synthesis of 1,3,5-triazine-based compounds starting from cyanuric chloride, and (b) structures of compounds used in practical applications. HNu = nucleophile.^5–10^

1,3,5-Triazine-based compounds are extensively used in fundamental research and practical applications, owing to their facile preparation from 2,4,6-trichloro-1,3,5-triazine (cyanuric chloride).^1–3^ In general, the synthesis of these compounds involves sequential nucleophilic aromatic substitution (S_N_Ar) reactions with identical or different nucleophiles (*e.g.* alcohols, amines, thiols, and Grignard reagents) (Fig. 1a).^2–4^ With amine nucleophiles, the substitution pattern can be controlled by the reaction temperature, since the substitution of chlorine by nitrogen reduces the reactivity at the other sites. Specifically, the monosubstitution reaction of cyanuric chloride with an amine takes place below 0 °C, whereas the second substitution occurs around room temperature, and the third substitution reaction requires heating above 60 °C. Thus, by controlling the temperature of each reaction step, the preparation of triply functionalised 1,3,5-triazine derivatives can be performed in one-pot.

Fig. 1b illustrates practical applications of some 1,3,5-triazine-based compounds. The 1,3,5-triazine scaffold is essential for the formulation of a number of herbicides (*e.g.* atrazine and ametryn),^5^ anticancer drugs (*e.g.* carborane–triazine conjugates),^6,7^ and adhesives (*e.g.* epoxy-melamine cross-linkers).^8^ In organic synthesis, 1,3,5-triazine derivatives are used as activating reagents for carboxylic acids (*e.g.* 4-(4,6-dimethoxy-1,3,5-triazin-2-yl)-4-methylmorpholinium chloride, DMTMM·Cl),^9^ and as ligands in catalysis (*e.g.* phosphine–triazine ligands).^10^

The 1,3,5-triazine scaffold has also been widely used as building block in supramolecular chemistry and polymer science for the construction of discrete monodisperse oligomers featuring distinct structural topologies: linear, branched, macrocyclic, and dendrimeric.^2,3^ These synthetic oligomers have interesting molecular recognition and self-assembly characteristics, often derived from hydrogen-bonding interactions involving 2,4,6-triamine-1,3,5-triazine (melamine) groups.^11^ Melamine units have also been used as the backbone in recognition-encoded melamine oligomers (REMO) that form sequence-selective duplexes in a similar manner to nucleic acids.^12^

In this review, we focus on monodisperse 1,3,5-triazine-based oligomers. We illustrate selected synthetic approaches used for the preparation of linear oligomers (section 2), macrocycles (section 3), branched oligomers (section 4), and dendrimers (section 5). In each section, we discuss the molecular recognition and self-assembly properties of these oligomers based on experimental studies conducted in solution, and we only refer to solid-state structures when necessary to clarify structural features.

## 2. Linear oligomers

1,3,5-Triazine-based linear oligomers have triazine residues incorporated either into the backbone as structural components or as side-chain recognition units appended to the backbone. In some cases, the triazine components of the backbone can also function as recognition units. In solution, the linear oligomers interact with identical or different compounds leading to the self-assembly of hydrogen-bonded complexes (*e.g.* homo- and heteroduplexes).

**Fig. 2:**
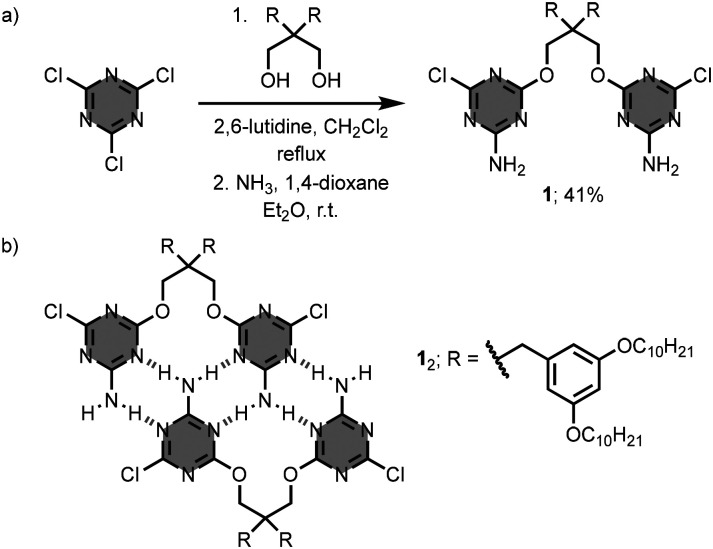
(a) Synthesis of 1,3,5-triazine-based linear oligomer 1, and (b) structure of the homoduplex 1_2_.^15,16^

Krische and co-workers proposed a synthetic approach, termed “covalent casting”, to prepare linear oligomers that can form homoduplexes.^13,14^ Specifically, the synthesis of the 2-mer oligomer 1 involves the connection of two molecules of cyanuric chloride with a 1,3-diol linker, followed by the treatment with ammonia (Fig. 2a).^15,16^ The 2-aminotriazine units in 1 can act as both hydrogen-bonding donor and acceptor groups. Thus, in chloroform solution, the oligomer 1 gives a homoduplex through the formation of 6 hydrogen-bonds (Fig. 2b). The relatively low self-association constant, *K*_a_ = 10^2^ M^−1^, suggests a geometry that is not optimal for self-assembly into the dimer 1_2_, probably, due to the steric and/or electronic clashes between the two oxygen atoms of the 1,3-diol linker.

**Fig. 3:**
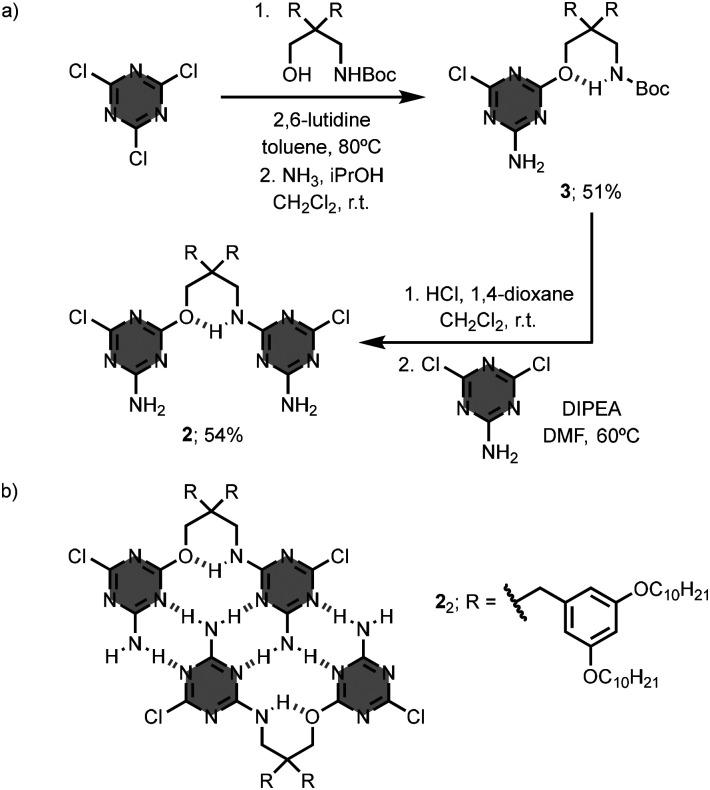
(a) Synthesis of 1,3,5-triazine-based linear oligomer 2, and (b) structure of the homoduplex 2_2_.^16,17^

To solve this issue, the authors replaced the 1,3-diol linker by a 1,3-amino alcohol in oligomer 2.^16,17^ The 1,3-amino alcohol linker establishes an intramolecular hydrogen-bond between the amino group and the oxygen atom that helps preorganise the structure of 2 for the formation of the homoduplex. The synthesis of the oligomer 2 involves the substitution of cyanuric chloride with the Boc-protected 1,3-amino alcohol linker and ammonia to give the intermediate 3, followed by cleavage of the Boc-protecting group and coupling with 2-amino-4,6-dichlorotriazine to obtain 2 (Fig. 3a). In chloroform solution, 2 self-assembles into the homoduplex 2_2_, with a self-association constant greater than 10^4^ M^−1^ (Fig. 3b). In 95 : 5 chloroform/dimethyl sulfoxide, the self-association constant for formation of 2_2_ is three orders of magnitude larger than that of 1_2_ (*K*_a_ = 2.8 × 10^4^ M^−1^*versus* 9.2 M^−1^). These results confirm that preorganisation of the linear oligomer has a significant impact on the thermodynamic stability of the duplex. Binding studies were carried out on a series of linear oligomers, and the stability of the homoduplex increases as the number of monomer units from one to three, indicating that the self-assembly process for duplex formation displays positive cooperativity. On the contrary, the binding free energy decreases for the homoduplex assembled from the 4-mer, which could be due to competitive binding processes, such as intramolecular folding.

**Fig. 4:**
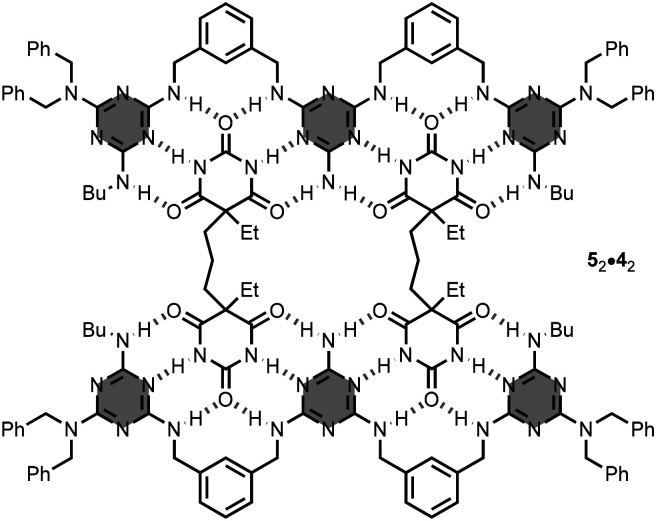
Structure of the 2 : 2 complex, 5_2_·4_2_.^18^

The research group of Timmerman and Reinhoudt described the 3-mer oligomer 4 having 2-aminotriazine units and *m*-xylylenediamine linkers in the backbone.^18,19^ In this case, the amino groups of the linkers can engage in intermolecular hydrogen-bonding interactions. The authors investigated the binding properties of the oligomer 4 with the bis-barbituric acid derivative 5. Interestingly, mixing equimolar amounts of 4 and 5 in chloroform solution leads to the self-assembly of a 2 : 2 complex, 5_2_·4_2_, stabilised by 24 hydrogen-bonds (Fig. 4). The formation of other species, such as the 1 : 1 complex, 5·4, or polymeric assemblies, (5·4)_*n*_, was not detected in the course of the titration experiment.

**Fig. 5:**
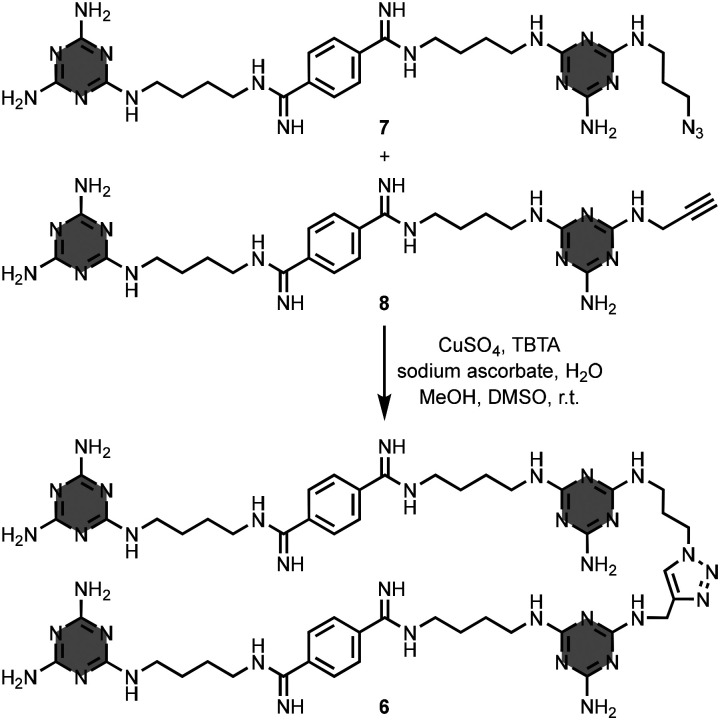
Synthesis of 1,3,5-triazine-based linear oligomer 6.^20^

Chan, Zimmerman and co-workers prepared the 4-mer oligomer 6 from the azide and alkyne linear precursors 7 and 8, using the copper(i)-catalysed azide–alkyne cycloaddition (CuAAC) reaction (Fig. 5).^20^ The oligomer 6 contains four melamine and two bis-amidinium units, connected by alkyl and triazole linkers, in the backbone. The authors showed that 6 interacts with nucleic acids and inhibits the formation of nucleic acid–protein complexes.

**Fig. 6:**
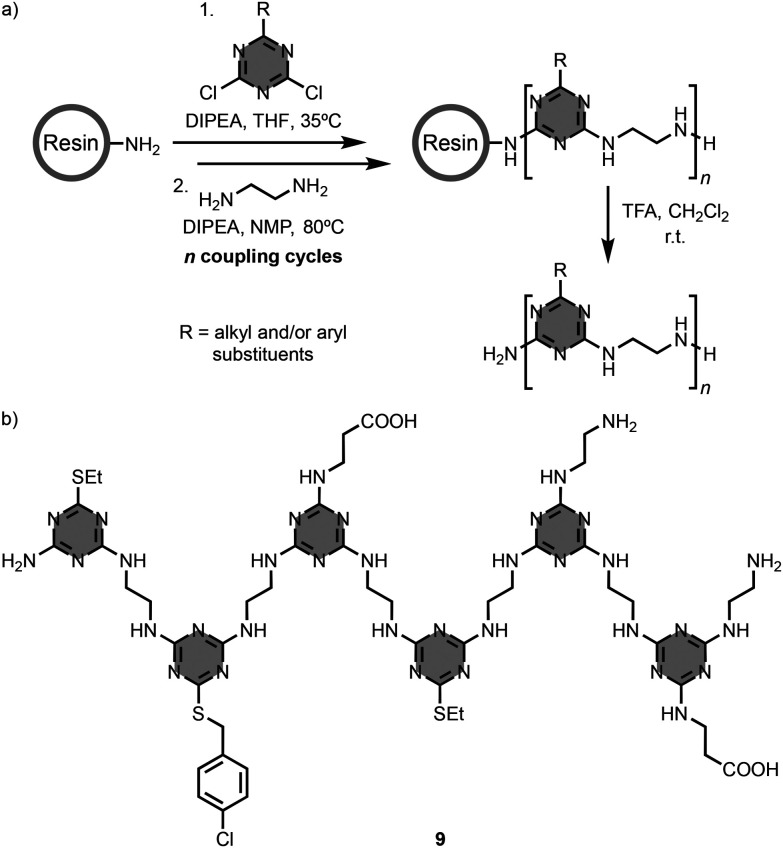
(a) Solid-phase synthesis of 1,3,5-triazine-based linear oligomers, and (b) structure of 9.^21^

Linear oligomers formed by a triazine backbone with information encoded as a sequence of different side-chains have also been described. For example, Grate and co-workers used solid-phase synthesis to prepare the 6-mer oligomer 9, which is decorated with a number of different side-chains (Fig. 6a and b).^21^ Starting from a resin with terminal amino groups, the synthetic approach consists of iterative S_N_Ar reactions between functionalised dichlorotriazine units and 1,2-ethylenediamine linkers. These linkers in the backbone provide conformational flexibility and sites for hydrogen-bonding interactions, but the supramolecular assembly properties of these systems were not characterised experimentally.^22,23^ Related oligomers were explored to assess their potential as therapeutic agents.^24^

**Fig. 7:**
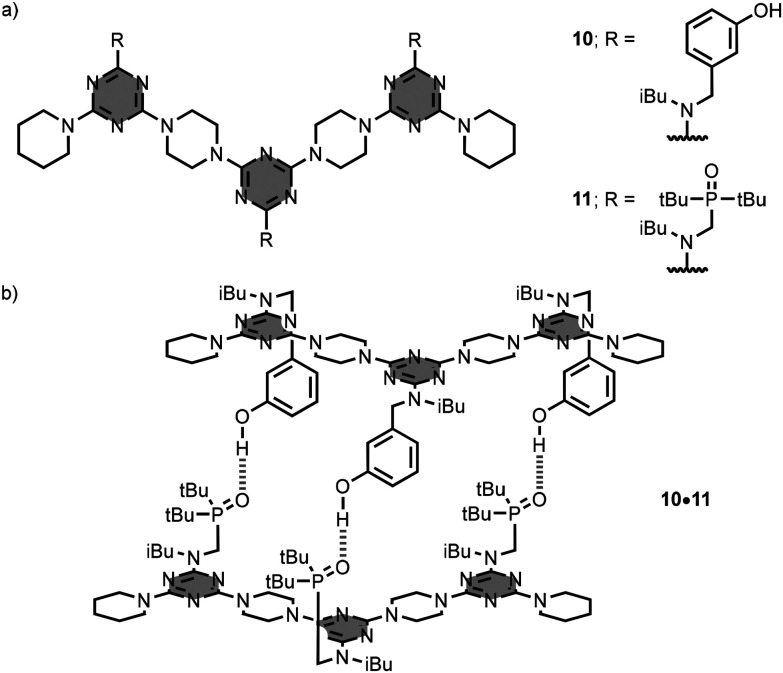
Structures of (a) 1,3,5-triazine-based linear recognition-encoded melamine oligomers (REMO) 10–11, and (b) heteroduplex 10·11.^25^

Hunter *et al*. have developed a series of linear oligomers featuring a triazine–piperazine backbone equipped with phenol and phosphine oxide side-chains (REMO).^25^ In this case, the nitrogen atoms of the triazine units in the backbone are sterically blocked by the piperazine linkers, so they cannot act as hydrogen-bonding acceptor groups. The side-chains appended to the backbone are the recognition units that encode the self-assembly of duplexes. For example, the complementary 3-mer oligomers 10 and 11 bear phenol and phosphine oxide recognition units, respectively (Fig. 7a). In toluene solution, an equimolar mixture of 10 and 11 gives an heteroduplex, stabilised by 3 hydrogen-bonding interactions (Fig. 7b). The heteroduplex 10·11 displays a remarkable thermodynamic stability with a *K*_a_ value of 1.6 × 10^5^ M^−1^, and positive cooperativity in the self-assembly process. In addition, the authors demonstrated that there are no intramolecular hydrogen-bonding interactions between the recognition units in mixed sequence 3-mers, which leads to high-fidelity sequence-selective duplex formation. The interaction of all possible 3-mer REMO sequences was investigated, and sequence-complementary duplexes were found to be an order of magnitude more stable than combinations with a single mismatch.

**Fig. 8:**
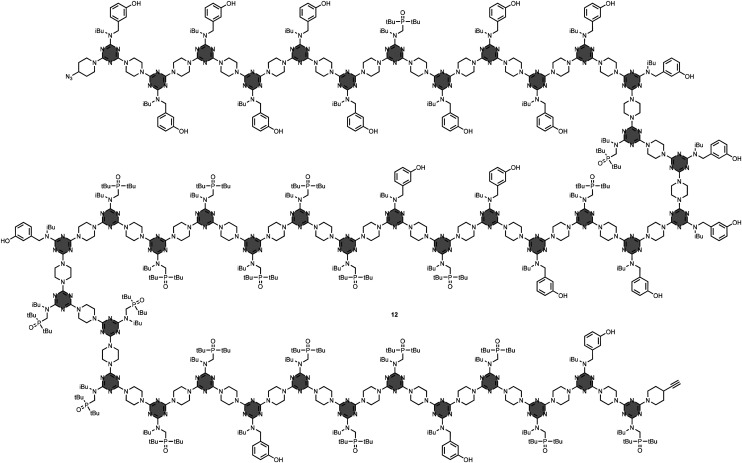
Structure of REMO 12 prepared by automated solid-phase synthesis.^26^

**Fig. 9:**
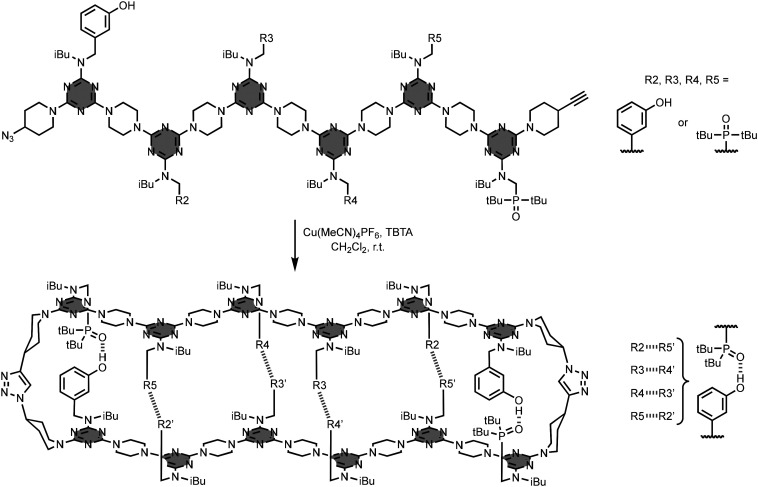
Trapping of REMO duplexes in a library of mixed sequence 6-mers. All possible combinations of the two recognition side-chains are present at positions R2–R5 on the oligomers.^27^

The preparation of REMO exploited iterative S_N_Ar reactions between functionalised dichlorotriazine units and piperazine linkers, and this process was automated using solid-phase synthesis on a peptide synthesiser.^26^ The longest sequence-defined synthetic oligomer reported to date is the 42-mer 12, which was obtained in excellent yield and purity by this method (Fig. 8). An usual feature of this synthetic route is that the reactivity of the dichlorotriazine monomers is independent of the nature of the recognition units on the side-chains, so it is possible to create randomised libraries of mixtures of different REMO sequences by simply mixing different dichlorotriazines in each coupling cycle on the synthesiser.^27^Fig. 9 shows a library of 6-mer sequences, which was prepared as a mixture of all possible phenol/phosphine oxide combinations at positions R2–R5 on the oligomer using this approach. The oligomers were equipped with a terminal azide at the phenol end and a terminal alkyne at the phosphine oxide end, so that a CuAAC reaction could be used to covalently trap any duplexes formed in the mixture. Since the sequence-complement of every REMO was present in the library, the only macrocyclic duplex products obtained were the combinations of oligomers containing a total of six phosphine oxide and six phenol side-chains.

**Fig. 10:**
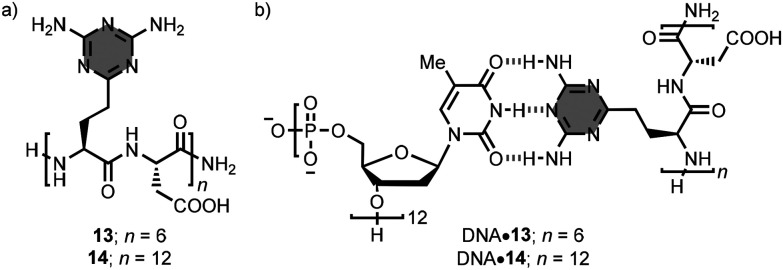
Structures of (a) 1,3,5-triazine-based linear oligomers 13–14, and (b) heteroduplexes DNA·13/14.^28^

Other examples of linear oligomers have triazine recognition units in the side-chains appended to the backbone. Krishnamurthy, Eschenmoser and co-workers prepared the 6- and 12-mer oligomers 13 and 14, respectively, using an automated solid-phase peptide synthesiser (Fig. 10a).^28^ These oligomers have 2,4-diaminotriazine units that can interact with the nucleobases of complementary DNA and RNA oligomers. For example, the combination of 13 with a 12-mer DNA strand, 5′-(dT)_12_-3′, in phosphate buffer at pH 7.0 afforded the heteroduplex, which was characterised by a melting temperature (*T*_m_) of 32 °C (Fig. 10b). Replacing the oligomer 13 by the longer counterpart 14 produced an enhancement of 22 °C in the *T*_m_ value due to the corresponding increase in the number of base-pairing interactions. Similar results were obtained with an RNA 12-mer.

**Fig. 11:**
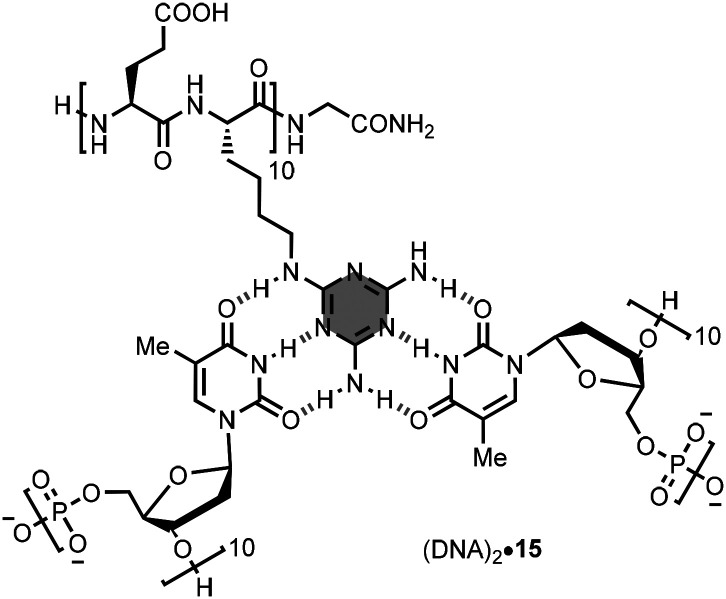
Structure of the 2 : 1 complex formed by 15 with DNA.^29^

Bong *et al*. synthesised oligomer 15, which contains ten melamine side-chains (Fig. 11).^29^ In phosphate buffer at pH 7.4, 15 formed a 2 : 1 complex with a 10-mer DNA strand, (dT)_10_. The 2 : 1 complex exhibited a melting temperature of 43 °C, which increased to 54 °C when the two DNA strands were covalently connected by a (dC)_10_ loop. The authors also showed that 15 can act as an allosteric effector of nucleic acid-based aptamers and ribozymes, activating binding or catalytic functions respectively.^30^ In addition, the interaction of 15 with nucleic acids inhibited enzymatic processes that required nucleic acid–protein association.^31^ Changing the peptide backbone to a peptoid resulted in less thermodynamically stable complexes.^32^

**Fig. 12:**
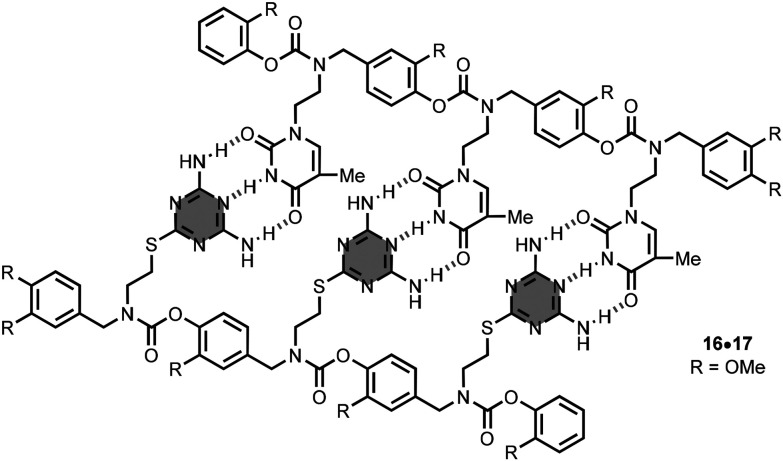
Structure of the heteroduplex 16·17.^33^

Alabi *et al*. synthesised the 3-mer oligomer 16, which has 2,4-diaminotriazine side-chains, by iterative reductive amination and carbamation reactions.^33^ The oligomer 16 formed an heteroduplex with the 3-mer oligomer 17, comprised of thymine nucleobases (Fig. 12). There are 9 intermolecular hydrogen-bonding interactions in the heteroduplex 16·17, and the association constant is 7.9 × 10^4^ M^−1^ in chloroform solution. Binding studies with a series of linear oligomers indicate positive cooperativity in the self-assembly process.

## 3. Macrocycles

1,3,5-Triazine-based macrocycles consist of triazine units linked together by either diamines or diols in a cyclic fashion. The macrocycles bearing hydrogen-bonding donor and acceptor groups can recognise complementary substrates, such as cyanuric acid, barbituric acid derivatives, and monosaccharides. The macrocycles can also bind mono- and polyatomic anions by establishing anion–π interactions with the triazine units.

**Fig. 13:**
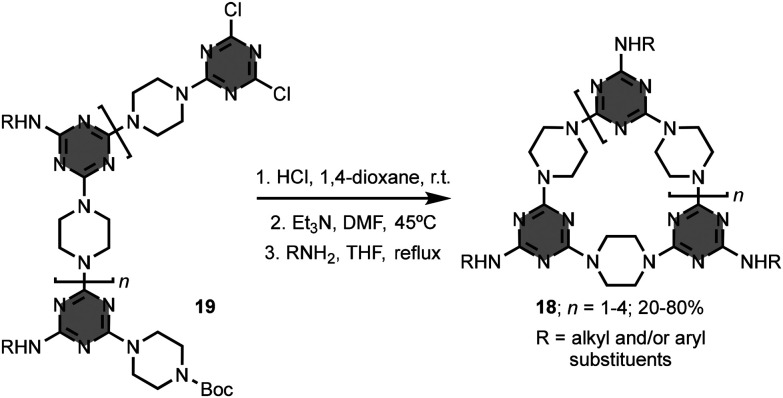
Synthesis of 1,3,5-triazine-based macrocycles 18.^34,35^

Typically, the synthesis of 1,3,5-triazine-based macrocycles requires the preparation of the linear precursor, followed by an intramolecular macrocyclisation reaction. Lowe and co-workers reported a series of macrocycles (*i.e.* 3-, 4-, 5-, and 6-mers) having piperazine linkers. Preparation of the macrocycles 18 from the linear precursors 19 involved removal of the Boc-protecting group of the terminal piperazine unit in 19, macrocyclisation, and substitution of the remaining chlorine atom with a primary amine (Fig. 13).^34,35^ Although these macrocycles have a bowl-shaped cavity, the molecular recognition properties were not investigated. It is worth mentioning that the preorganisation of the terminal reacting groups by intramolecular hydrogen-bonds in the linear precursor can lead to excellent yields in the macrocyclisation reaction.^36–38^

**Fig. 14:**
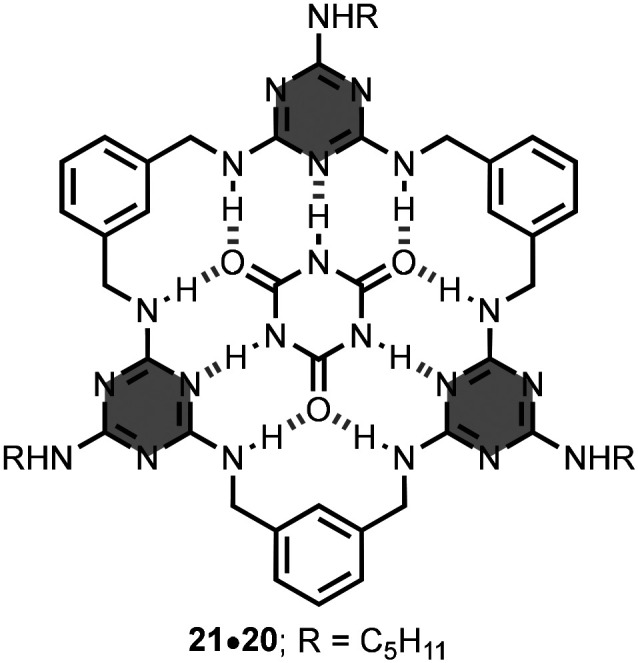
Structure of the 21·20 complex.^35^

The research group of Lowe prepared macrocycle 20 using *m*-xylylenediamine linkers.^35^ The structure of 20 has an array of hydrogen-bond donors and acceptors inwardly-directed into the central cavity. Excellent complementarity with cyanuric acid 21 was found with possibility for 9 intermolecular hydrogen-bonds (Fig. 14). Addition of 21 to 20 leads to the formation of a 1 : 1 complex, 21·20, with an association constant of 2.5 × 10^4^ M^−1^ in chloroform solution. Macrocycle 20 is also capable of binding octyl glycosides in the same solvent.^35^ These complexes have a 1 : 1 stoichiometry and association constants in the range 10^3^–10^4^ M^−1^. Replacing the *m*-xylylenediamine linkers by *m*-phenylenediamine counterparts in the macrocycle prevented the binding with the same substrates.^39^

Kondo and co-workers employed a similar macrocycle with *m*-xylylenediamine linkers to study complexation of barbital.^40^ In this case, 1 : 1 and 2 : 1 complexes were observed in chloroform solution. The association constants, *K*_1:1_ = 4.2 × 10^4^ M^−1^ and *K*_2:1_ = 2.4 × 10^2^ M^−1^, indicate negative cooperativity for the binding of the second guest molecule. An X-ray crystal structure of the 2 : 1 complex showed that one barbital molecule binds to two of the melamine units through 6 hydrogen-bonding interactions, and the other barbital molecule forms 3 hydrogen-bonds with the remaining melamine unit.

**Fig. 15:**
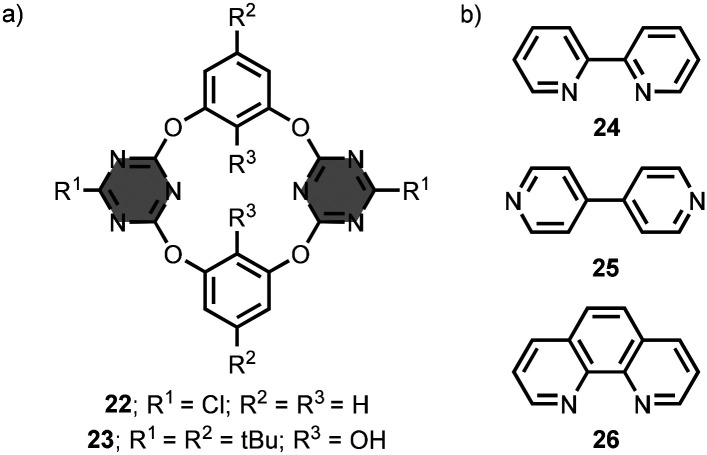
Structures of (a) 1,3,5-triazine-based macrocycles 22–23, and (b) substrates 24–26.^47–49,51^

Wang *et al*. developed a series of oxygen-bridged calix[2]arene[2]triazine macrocycles.^41–46^ These macrocycles are fixed in a 1,3-alternate conformation and possess a cleft-like cavity. For example, the macrocycle 22 contains two opposing monochlorotriazine units connected by 1,3-benzenediol linkers (Fig. 15a).^47^ In acetonitrile solution, the macrocycle 22 forms 1 : 1 complexes with anions, such as chloride, nitrate and hexafluorophosphate, with association constants between 10^2^ and 10^4^ M^−1^.^48,49^ In these complexes, the substrates make anion–π interactions with the receptor. The attachment of an aza-crown ether moiety at the R^2^ positions gave ion-pair receptors.^50^ Functionalisation of the R^3^ positions with hydroxyl groups in 23 enabled the binding of neutral substrates, such as 2,2′-bipyridine 24, 4,4′-bipyridine 25 and 1,10-phenanthroline 26, *via* hydrogen-bonding interactions (Fig. 15a and b).^51^ The association constants for formation of 1 : 1 complexes are between 10 and 10^2^ M^−1^ in chloroform solution. Structurally related macrocycles were used as receptors in enantioselective molecular recognition and were incorporated into lipid membranes to act as ion-pair carriers.^52–56^

**Fig. 16:**
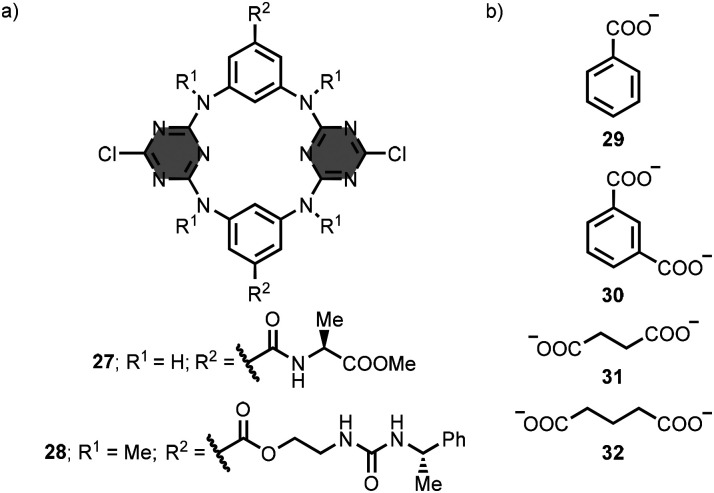
Structures of (a) 1,3,5-triazine-based macrocycles 27–28, and (b) substrates 29–32 (used as tetrabutylammonium salts).^58,59^

Calix[2]arene[2]triazine macrocycles with nitrogen bridges were also reported.^57^ Moiteiro, Félix and co-workers decorated the macrocycles 27 and 28 with amide or urea groups at the R^2^ positions (Fig. 16a).^58,59^ Macrocycle 27 bound aromatic carboxylate anions in dimethyl sulfoxide solution. The association constants of the 1 : 1 anionic complexes formed with benzoate 29 and isophthalate 30 are between 10 and 10^3^ M^−1^ (Fig. 16b). Macrocycle 28 formed ion-pair complexes with the tetraalkylammonium salts of aliphatic carboxylate anions in chloroform solution. The association constants of the complexes formed with tetrabutylammonium succinate 31 and glutarate 32 are between 10^2^ and 10^3^ M^−1^ (Fig. 16b). In addition, the authors described a calix[2]arene[2]triazine macrocycle with carboxylate groups at R^1^ for the coordination of Cu(ii) ions.^60^

**Fig. 17:**
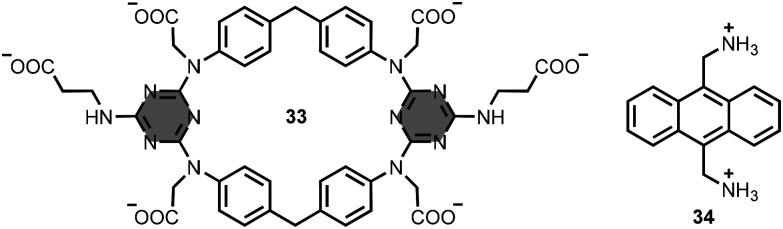
Structures of 1,3,5-triazine-based macrocycle 33 and substrate 34 (used as sodium and chloride salts, respectively).^61^

Kusano, Hayashida and co-workers reported macrocycle 33, which has an aromatic cavity defined by two diphenylmethane and two triazine units (Fig. 17).^61^ The macrocycle 33 also bears six carboxylate groups to ensure water solubility. In buffer solution at pH 7.4, addition of the diammonium anthracene derivative 34 (Fig. 17) gave a 1 : 1 complex with the anthracene moiety included within the hydrophobic aromatic cavity of 33, leading to an association constant between 10^3^ and 10^4^ M^−1^.

**Fig. 18:**
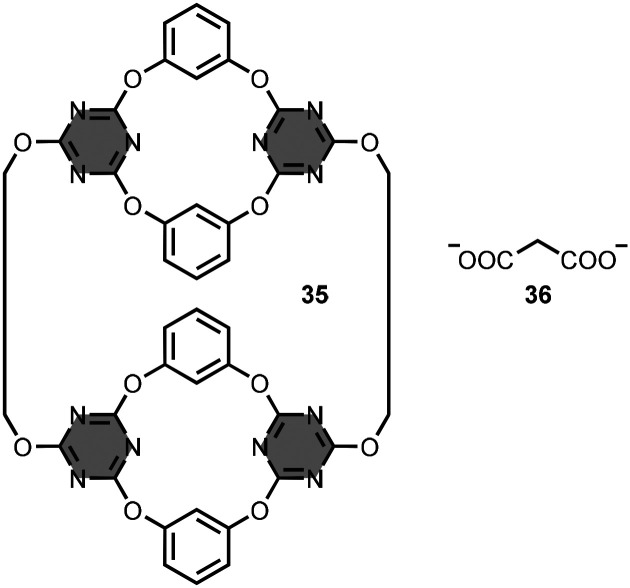
Structures of 1,3,5-triazine-based macrocycle 35 and substrate 36 (used as tetrabutylammonium salt).^67^

Triazine macrocycles with larger cavities have been described.^62–66^ Wang *et al*. prepared the bis-calix[2]arene[2]triazine macrocycle 35 (Fig. 18).^67^ Binding studies in chloroform solution showed low affinity interactions (association constants less than 10 M^−1^) with tetrabutylammonium salts of carboxylate anions, such as 2-carboxyacetate 36 (Fig. 18).

**Fig. 19:**
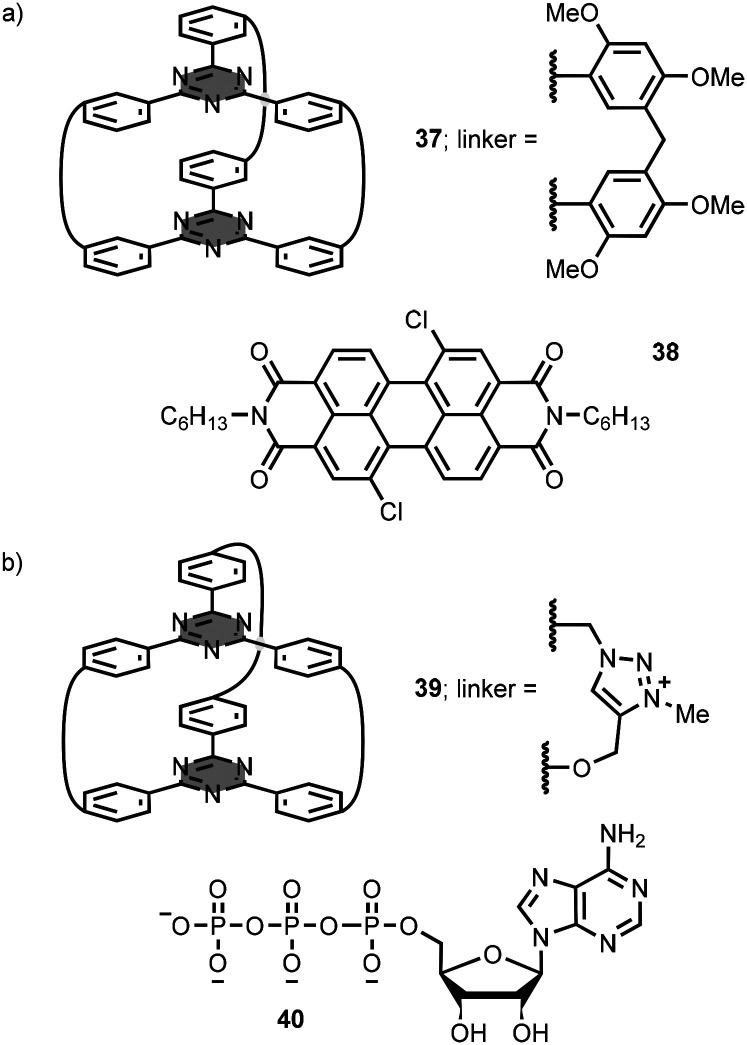
Structures of (a) macrocycle 37 and substrate 38, and (b) macrocycle 39 and substrate 40 (used as chloride and sodium salts, respectively).^68,69^

Macrobicycle 37 is based on two 1,3,5-triazine-based aromatic panels bridged by three diphenylmethanes (Fig. 19a).^68^ This architecture provides an aromatic cavity suitable for the inclusion of planar aromatic substrates, such as 38 (Fig. 19a). Association constants were measured in a range of different solvents with values as high as 10^5^ M^−1^ in dimethyl sulfoxide solution.

Natarajan *et al*. synthesised a similar macrobicycle 39 using a CuAAC reaction followed by methylation of the triazoles (Fig. 19b).^69^ The water-soluble tricationic receptor exhibits binding selectivity for adenosine-5′-triphosphate 40 (Fig. 19b) with an association constant of 10^4^ M^−1^ in aqueous buffer at pH 7.4.

## 4. Branched oligomers

1,3,5-Triazine-based branched oligomers have been prepared with a central hub and spokes containing melamine residues as recognition units. In solution, these branched oligomers form hydrogen-bonded assemblies, called rosettes, with either isocyanuric or barbituric acid derivatives.

**Fig. 20:**
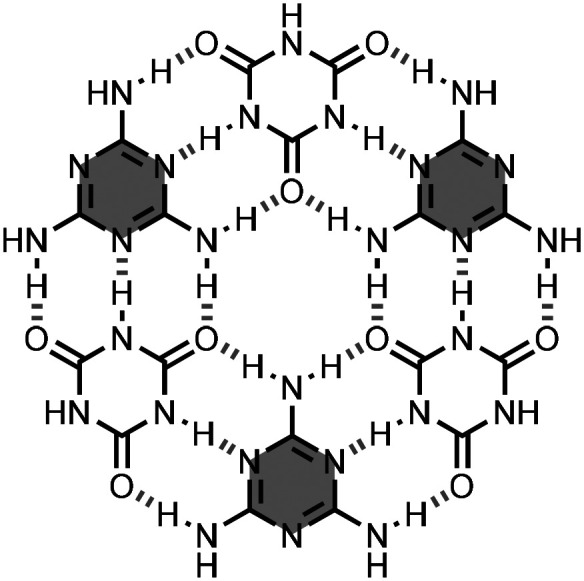
Structure of melamine·cyanuric acid rosette.^3,70^

Previous studies showed that the combination of melamine with isocyanuric acid in a 1 : 1 ratio produces a 2D hydrogen-bonded network.^3^ This 2D network contains the hydrogen-bonded rosette motif shown in Fig. 20.^70–72^ To control the formation of these rosettes in solution, one approach is preorganisation of the three melamine units using branched oligomers.

**Fig. 21:**
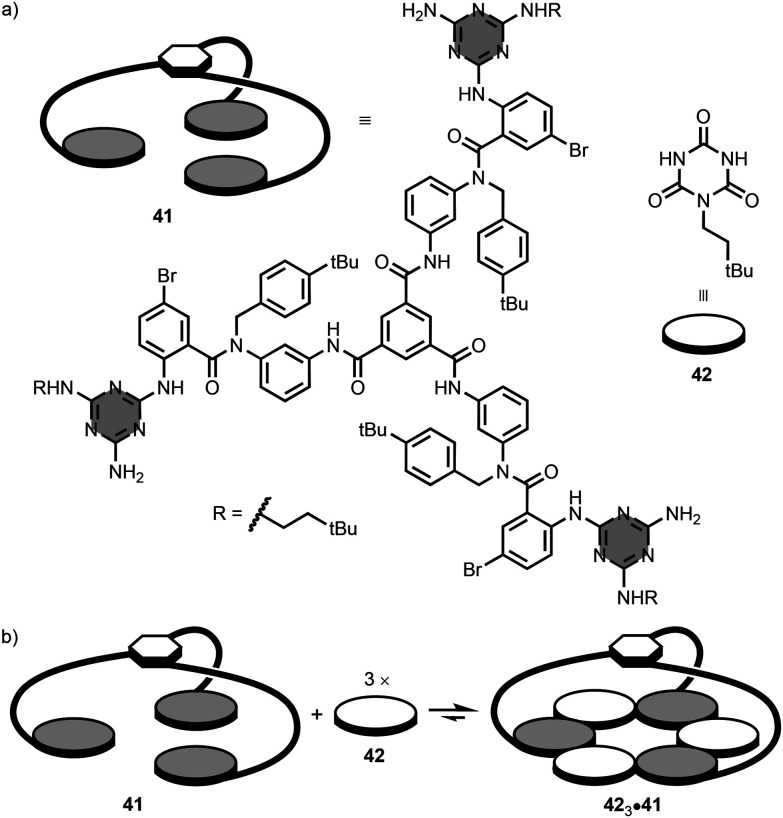
(a) Structures of 1,3,5-triazine-based branched oligomer 41 and isocyanuric acid derivative 42, and (b) self-assembly of the 3 : 1 complex, 42_3_·41.^74,75^

Whitesides and co-workers reported a series of branched melamine oligomers, and studied their self-assembly properties with isocyanuric acid derivatives.^73^ The synthesis of the branched oligomers involved reaction of 1,3,5-benzenetricarbonyl trichloride (*i.e.* central hub) with 3 equivalents of a linear 1,3,5-triazine-based oligomer functionalised with a primary amine (*i.e.* spoke). For example, the branched oligomer 41 has aromatic amide linkers that connect the three terminal melamine units to the central hub (Fig. 21a).^74–77^ Addition of *neo*-hexyl isocyanurate 42 to 41 leads to the 3 : 1 complex, 42_3_·41, in chloroform solution (Fig. 21b). The complex contains one rosette stabilised by 18 hydrogen-bonding interactions between the three melamine units of 41 and the three molecules of 42. It is worth mentioning that intermediate complexes (*i.e.* 1 : 1 and 2 : 1) were not detected in the titration, indicating that the self-assembly process displays positive cooperativity.

Bong *et al*. designed a similar branched oligomer with three terminal melamine units attached to a phospholipid central hub through alkyl amide linkers.^78,79^ This oligomer was incorporated into the lipid membranes of vesicles, and an analogue bearing three terminal isocyanurate units was incorporated into separate vesicles. In phosphate buffer at pH 6.7–7.4, the formation of a rosette involving the complementary branched oligomers promoted the interaction between the two types of vesicles. A similar approach was used for the binding of peptides to lipid membranes and the establishment of protein–protein assemblies.^80,81^

**Fig. 22:**
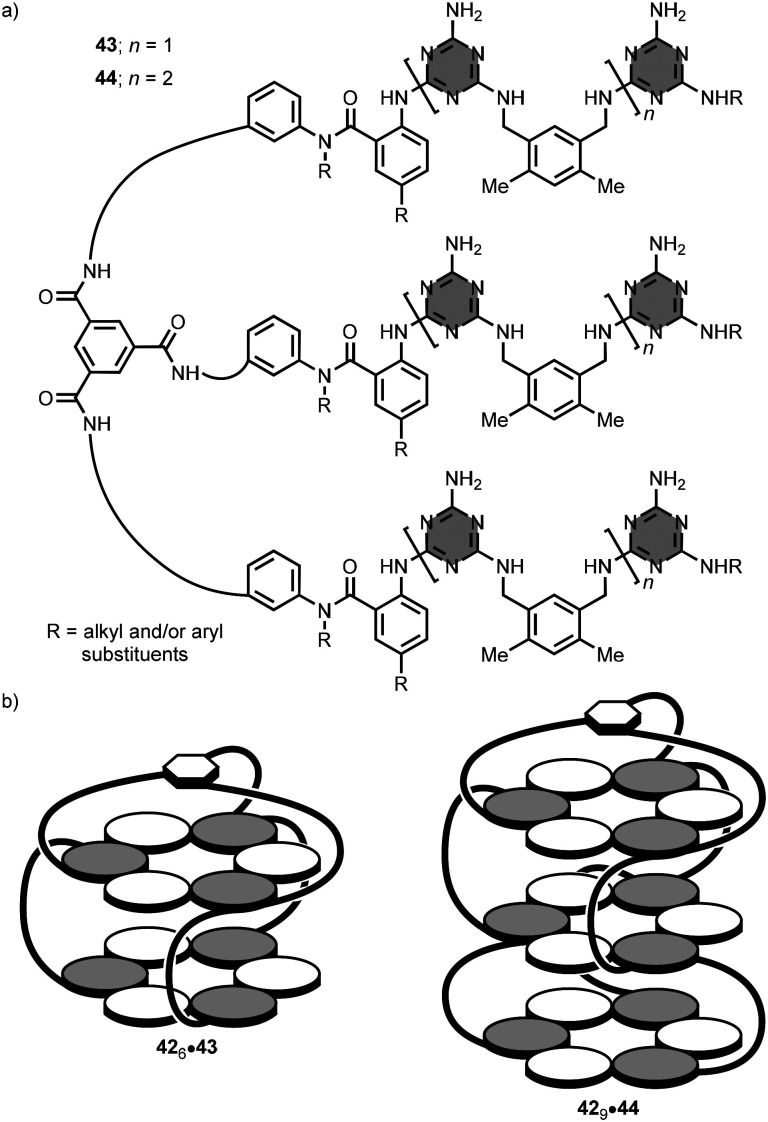
Structures of (a) 1,3,5-triazine-based branched oligomers 43–44, and (b) 42_6_·43 and 42_9_·44 complexes.^82,85^

The research group of Whitesides also increased the number of melamine units incorporated into the branched oligomers. Branched oligomer 43 has six melamine units: three are located at internal positions and the other three at terminal positions, separated by *m*-xylyl linkers (Fig. 22a).^82^ In the presence of 42, a 6 : 1 complex is formed with positive cooperativity in chloroform solution (Fig. 22b). The 42_6_·43 complex has two stacked rosettes with a total of 36 hydrogen-bonding interactions. The combination of bis-isocyanurate derivatives with either 41 or 43 also yielded assemblies featuring two stacked rosettes.^82–84^ Similarly, branched oligomer 44, which has nine melamine units, formed three stacked rosettes with nine molecules of 42 (Fig. 22a and b).^85^ The resulting 42_9_·44 complex is held together by a total of 54 hydrogen-bonding interactions.

**Fig. 23:**
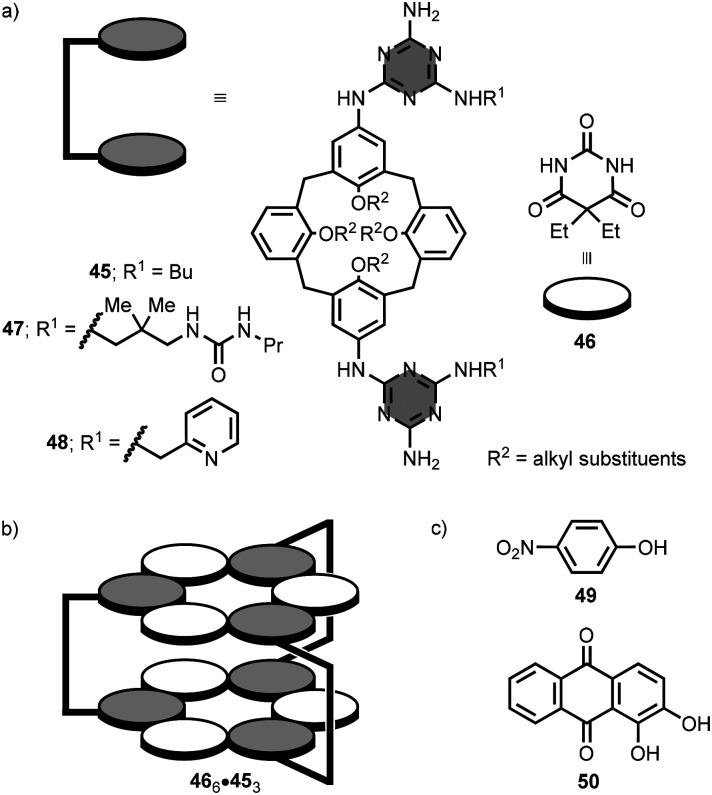
Structures of (a) 1,3,5-triazine-based calix[4]arene macrocycles 45, 47 and 48 and barbituric acid derivative 46, (b) 46_6_·45_3_ complex, and (c) substrates 49 and 50.^90–92^

A number of other research groups have used either macrocycles or linear oligomers to preorganise melamine units for the self-assembly of stacked rosettes. Reinhoudt and co-workers employed a series of calix[4]arene macrocycles with melamine units at the upper rim in combination with barbituric acid derivatives.^86–89^ For example, the calix[4]arene macrocycle 45 features two opposing melamine units decorated with *n*-butyl groups (Fig. 23a).^90^ The addition of 5,5-diethylbarbituric acid 46 to a chloroform solution of 45 gave the 6 : 3 complex, 46_6_·45_3_, containing two stacked rosettes (Fig. 23b). Replacing the *n*-butyl groups on the melamine units by urea or pyridyl groups in 47 and 48, respectively (Fig. 23a), endows the two stacked rosettes with additional molecular recognition properties.^91,92^ Both 46_6_·47_3_ and 46_6_·48_3_ can interact with polar aromatic substrates in chloroform solution. While six molecules of 4-nitrophenol 49 bind at the periphery of 46_6_·47_3_, three molecules of alizarin 50 (Fig. 23c) are encapsulated within 46_6_·48_3_*via* hydrogen-bonding and aromatic stacking interactions.

**Fig. 24:**
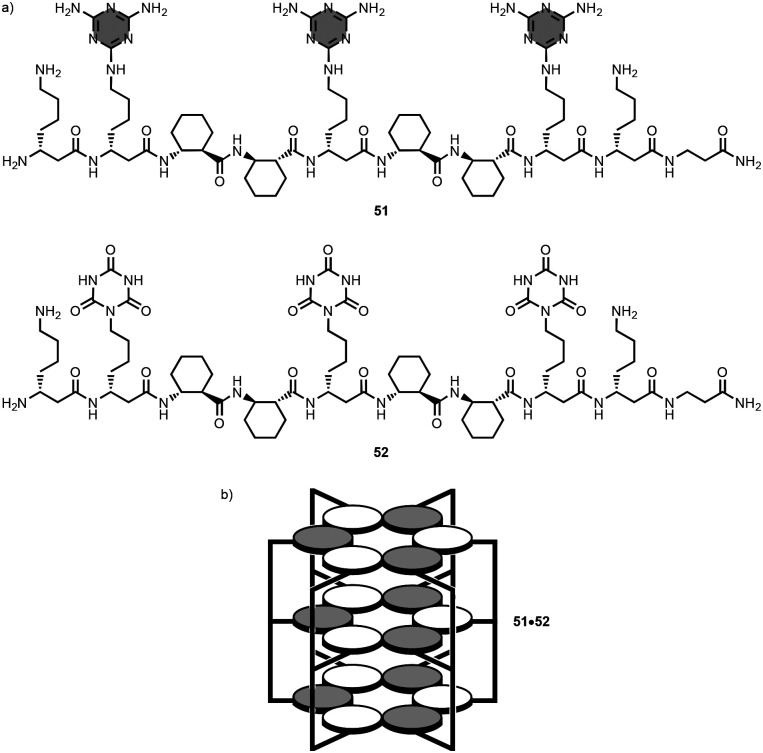
Structures of (a) 1,3,5-triazine-based linear oligomers 51–52, and (b) 51·52 complex.^93^

Diederichsen *et al*. prepared the 10-mer linear oligomers 51 and 52 featuring three melamine and three isocyanurate side-chains, respectively, using an automated solid-phase peptide synthesiser (Fig. 24a).^93^ In acetate buffer at pH 7.4, the peptide backbone of 51 and 52 adopt a right-handed helical conformation that arranges the complementary recognition units for self-assembly of three stacked rosettes (Fig. 24b).

## 5. Dendrimers

1,3,5-Triazine-based dendrimers are comprised of melamine branching units linked to a central core.^94,95^ The branching units possess terminal functional groups exposed at the surface of the dendrimer. The number of branching points defines the dendrimer generation (*i.e.* G*n*, where *n* is the generation number), which determines the overall size and the number of terminal groups. In aqueous solution, 1,3,5-triazine-based dendrimers adopt globular three-dimensional structures, and have been found to bind hydrophobic compounds.

**Fig. 25:**
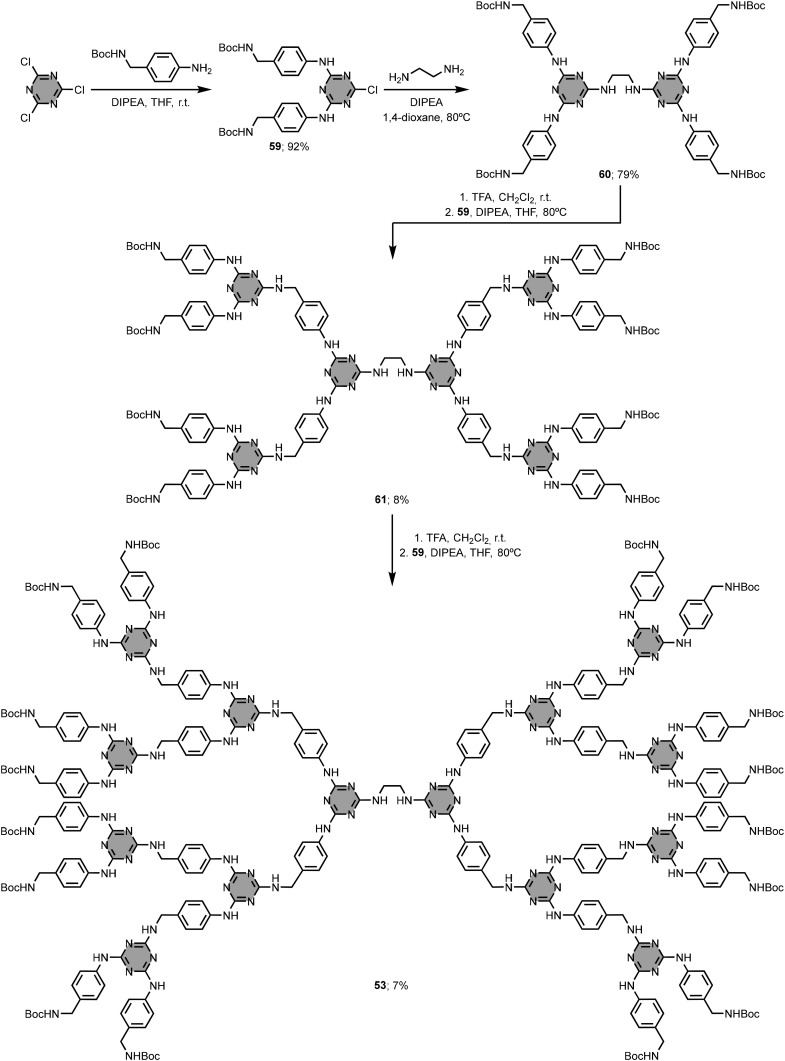
Divergent synthesis of 1,3,5-triazine-based dendrimer 53.^102^

**Fig. 26:**
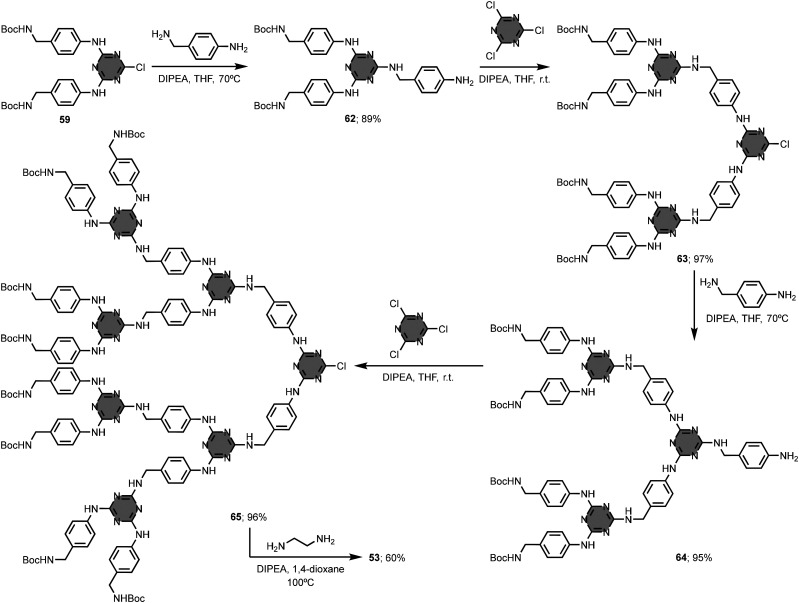
Convergent synthesis of 1,3,5-triazine-based dendrimer 53.^102^

In general, the construction of 1,3,5-triazine-based dendrimers follows either a divergent or a convergent synthetic approach.^96–101^ While the divergent approach consists of synthesising the dendrimer from the central core to the periphery, the convergent approach involves the preparation of the branching units (dendrons) that are then attached to the central core. Simanek and co-workers compared the two synthetic approaches for the preparation of the third generation (G3) dendrimer 53.^102^ This compound has a 1,2-ethylenediamine unit as central core, melamine groups connected by 4-aminobenzylamine linkers as branching units, and 16 Boc-protected amino groups at the periphery. The divergent approach shown in Fig. 25 uses sequential coupling and deprotection of the monoprotected diamine linker, but the isolated overall yield of 53 was less than 1%. The convergent approach gave the G3 dendrimer 53 in good yield (47%) and excellent purity (Fig. 26). Although the divergent approach was used to synthesise larger analogues (*e.g.* the G13 dendrimer),^103,104^ it becomes increasingly difficult to identify defects and separate side-products after each coupling cycle when *n* is greater than 3, and the overall yields are very low. These issues can be alleviated to some extent using more elaborate monomers (macromonomers).^105^ In addition to the high yields, an attractive feature of the convergent approach is that protecting groups are not required on the diamine linkers, provided the two amino groups exhibit sufficiently different reactivities.^106,107^ However, the convergent approach is limited to the synthesis of dendrimers up to G3, since the yield of the final reaction step involving the central core is limited by steric clashes between the large dendrons.

**Fig. 27:**
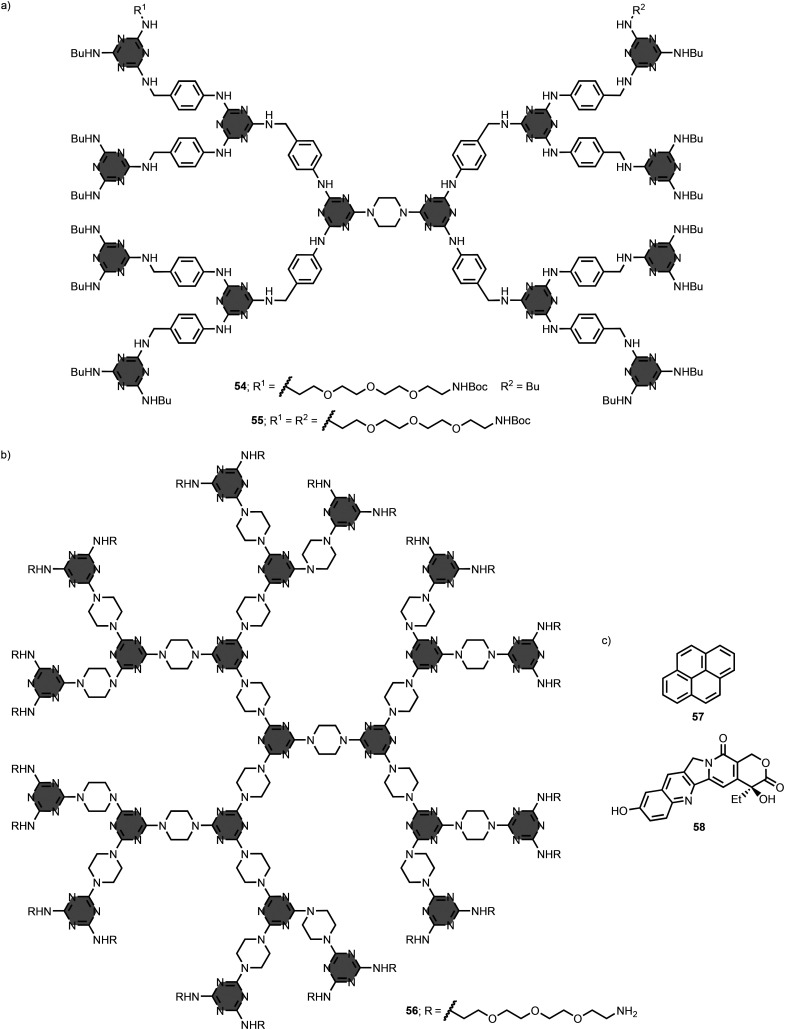
Structures of 1,3,5-triazine-based dendrimers (a) 54–55 and (b) 56, and (c) substrates 57 and 58.^111,112^

Simanek *et al*. described methods for functionalisation of the peripheral groups on the dendrimers.^108,109^ Incorporation of 48 ionisable groups (*e.g.* amine, guanidine or carboxylate) on the ends of the arms of G3 dendrimers resulted in good solubilities in water.^110^ It was also possible to selectively functionalise G3 dendrimers with either one or two polyethylene glycol chains bearing Boc-protected amino groups (compounds 54 and 55 in Fig. 27a).^111^ These dendrimers have *n*-butyl groups at the remaining terminal positions.

The research group of Simanek applied dendrimers to the solubilisation of hydrophobic substrates in aqueous solution. Specifically, the G3 dendrimer 56, equipped with 24 polyethylene glycol chains and terminal amino groups, is capable of binding and increasing the solubilities of pyrene 57 and 10-hydroxycamptothecin 58 in phosphate buffer at pH 7.5 (Fig. 27b and c).^112^ Using a series of odd generation dendrimers having polyethylene glycol and piperazine linkers, the authors showed that the number of bound hydrophobic substrates increases with the dendrimer generation until G7, but decreases at G9.^113^ Based on these findings, preliminary studies were conducted to evaluate the use of 1,3,5-triazine-based dendrimers as drug carriers.^114^

## 6. Conclusion and perspectives

In summary, a variety of different types of monodisperse 1,3,5-triazine-based oligomers have been explored featuring distinct structural topologies. Linear oligomers have been prepared by iterative S_N_Ar reactions in solution or using solid-phase synthesis. The incorporation of recognition units either into the backbone or into the side-chains encodes the formation of hydrogen-bonded duplexes, which resemble the duplex structures of nucleic acids. Intramolecular S_N_Ar reactions of linear oligomers has been used to obtain the corresponding macrocycles. These cyclic oligomers are capable of binding complementary substrates. Branched oligomers have been synthesised by the attachment of linear oligomers to a central core. In the presence of complementary isocyanuric or barbituric acid derivatives, branched melamine oligomers self-assemble into hydrogen-bonded rosettes. Finally, the dendrimers with branching units linked to a central core have been prepared by either divergent or convergent synthetic approaches involving S_N_Ar reactions. The dendrimers adopt globular three-dimensional structures in aqueous solution and can encapsulate hydrophobic substrates in their interior. The synthetic accessibility of monodisperse 1,3,5-triazine-based oligomers makes them an attractive target for applications in different areas of research, such as in supramolecular chemistry, polymer science, materials science, and chemical biology, and the advent of automated synthesis opens the way for rapid development of these platforms.

## Author contributions

Conceptualisation, L. E. and C. A. H.; funding acquisition, C. A. H.; writing – original draft, L. E.; writing – review and editing, L. E. and C. A. H.

## Data availability

This is a review article that does not report original data.

## Conflicts of interest

There are no conflicts to declare.
